# Comparison of emissions across tobacco products: A slippery slope in tobacco control

**DOI:** 10.18332/tid/183797

**Published:** 2024-03-30

**Authors:** Ahmad El-Hellani, Ayomipo Adeniji, Hanno C. Erythropel, Qixin Wang, Thomas Lamb, Vladimir B. Mikheev, Irfan Rahman, Irina Stepanov, Robert M. Strongin, Theodore L. Wagener, Marielle C. Brinkman

**Affiliations:** 1 Division of Environmental Health Sciences, College of Public Health, The Ohio State University, Columbus, United States; 2 Center for Tobacco Research, The Ohio State University Comprehensive Cancer Center, Columbus, United States; 3 Department of Chemical and Environmental Engineering, School of Engineering & Applied Science, Yale University, New Haven, United States; 4 Yale Center for the Study of Tobacco Product Use and Addiction (YCSTP), Department of Psychiatry, Yale School of Medicine, New Haven, United States; 5 Department of Environmental Medicine, University of Rochester Medical Center, Rochester, United States; 6 Battelle Public Health Center for Tobacco Research, Battelle Memorial Institute, Columbus, United States; 7 Division of Environmental Health Sciences, School of Public Health, University of Minnesota Twin Cities, Minneapolis, United States; 8 Masonic Cancer Center, University of Minnesota Twin Cities, Minneapolis, United States; 9 Department of Chemistry, Portland State University, Portland, United States; 10 Department of Internal Medicine, The Ohio State University, Columbus, United States; 11 Division of Epidemiology, College of Public Health, The Ohio State University, Columbus, United States

**Keywords:** tobacco products, emission comparison, sample generation, analytical methods, toxicants

## Abstract

In this narrative review, we highlight the challenges of comparing emissions from different tobacco products under controlled laboratory settings (using smoking/vaping machines). We focus on tobacco products that generate inhalable smoke or aerosol, such as cigarettes, cigars, hookah, electronic cigarettes, and heated tobacco products. We discuss challenges associated with sample generation including variability of smoking/vaping machines, lack of standardized adaptors that connect smoking/vaping machines to different tobacco products, puffing protocols that are not representative of actual use, and sample generation session length (minutes or number of puffs) that depends on product characteristics. We also discuss the challenges of physically characterizing and trapping emissions from products with different aerosol characteristics. Challenges to analytical method development are also covered, highlighting matrix effects, order of magnitude differences in analyte levels, and the necessity of tailored quality control/quality assurance measures. The review highlights two approaches in selecting emissions to monitor across products, one focusing on toxicants that were detected and quantified with optimized methods for combustible cigarettes, and the other looking for product-specific toxicants using non-targeted analysis. The challenges of data reporting and statistical analysis that allow meaningful comparison across products are also discussed. We end the review by highlighting that even if the technical challenges are overcome, emission comparison may obscure the absolute exposure from novel products if we only focus on relative exposure compared to combustible products.

## INTRODUCTION

The global tobacco product landscape has changed immensely during the last few decades with many new products promoted with reduced exposure and risk claims^1,2^. Tobacco products are categorized as inhalable products that generate an inhalable aerosol/smoke or smokeless tobacco products that are consumed by placing the product between the gum and cheek or lip or by sniffing (e.g. oral nicotine pouches or dry snuff). Inhalable tobacco products are further categorized into self-sustained combustion products (e.g. cigarettes and cigars), assisted-combustion products (e.g. hookah), and electronic products [e.g. heated tobacco products (HTPs) and a wide range of electronic cigarettes (ECs)]. This ever-growing landscape complicates tobacco control especially as stakeholders including regulators, scientists, industry, consumers, and the general public engage in comparing tobacco products focusing on the relative risk, rather than the absolute risk of new products^3^. Looking at the relative risk of newly introduced tobacco products could be misleading as it may not account for the unique complexities and toxicities of these products. Comparing the risks of inhalable tobacco products necessitates a comparison of their emissions, yet this comparison is not straightforward due to technical challenges as we aim to show in this article.

Several international, federal, and local jurisdictions, including the US Food and Drug Administration (FDA), have introduced regulatory mechanisms that rely mainly on the comparison of tobacco product emissions and risks. For example, one way to obtain authorization from the US FDA for a Modified Risk Tobacco Product claim would be to demonstrate that under normal consumer use, a novel tobacco product exposes users to fewer and lower levels of toxicants compared to the exposures from their usual tobacco product^4^. Exposure to toxicants from tobacco product use is typically studied using *in vitro* and *in vivo* models, as well as via biomarkers of exposure in human studies that rely on user behavior (including intensity, duration, and frequency) and product emissions^5^. The focus of this article is on the comparison of inhalable tobacco product emissions assessed in a controlled laboratory setting using a smoking/vaping machine for sample generation^6^.

Comparison of tobacco product emissions dates back to the introduction of filtered cigarettes when independent and industry-affiliated researchers engaged in comparing their emissions to those of unfiltered cigarettes^7^. This practice continued in the decades that followed, comparing emissions from filtered cigarettes with different filter ventilation^8^, cigars to cigarettes^9^, hookah to cigarettes^10^, and newly introduced ECs and HTPs to cigarettes^11-13^. Although inter-brand comparisons within the same product category have been conducted for decades, there is no standard intra- or inter-brand comparison methodology^14^. Indeed, the challenges of comparing emissions from tobacco products go beyond inter-laboratory and intra-laboratory variabilities to the roots of the validity and feasibility of this comparison. These challenges include the conditions of sample generation (i.e. smoking machine parameters and product conditioning before testing), the efficiency and suitability of emission trapping (i.e. different methods to trap the particle and gas phases of the smoke/aerosol, different techniques, and storage conditions before analysis), the considerations of analytical methods (i.e. sensitivity, precision, repeatability, and reproducibility), selection of emissions to monitor (i.e. assessing common toxicants across products and determining product-specific toxicants), and data reporting (e.g. quantitation per product, per mass or volume, per puff, per nicotine yield, etc.). A discussion of these challenges will be the focus of this review (Table 1).

**Table 1:** A summary of the main challenges in comparing emissions across tobacco products and some recommendations to standardize the comparison

*Challenges*	*Recommendations*
**Sample generation**	Use the same smoking/vaping machine to conduct testing across products if possible. Cross-validate different smoking/vaping machines if needed. Develop universal leak-free smoking/vaping machine adaptors that fit different tobacco product mouth ends.
**Puffing conditions**	Report all puffing/sampling conditions. Develop product-specific puffing regimes using data collected from clinical and epidemiological studies. Standardize the smoking/vaping session length across products based on a common parameter (e.g. nicotine yield).
**Product-specific sampling conditions**	Mimic actual use of products when testing in the lab (e.g. preheating). Develop reference products for all types of tobacco products.
**Techniques of aerosol trapping**	Optimize aerosol/smoke trapping methods to account for the specificity of each product (e.g. liquid or solid particles, difference in analyte levels across products, and partitioning of analytes between particle and gas phases). Cross-validate different trapping methods if needed.
**Analytical method suitability**	Optimize analytical methods to account for different matrices and constituent levels across products. Cross-validate different analytical methods if needed.
**Particle size distribution**	Optimize analytical methods and techniques used to characterize particles in the aerosol of different tobacco products. Optimize the conditions to collect particles from different products with minimum perturbance of the particles. Cross-validate different particle size distribution methods if needed.
**Toxicants to monitor**	Update the priority lists of toxicants to reflect exposure from new and emerging products (e.g. the FDA HPHC list). Generate more inhalation toxicity data on compounds found in tobacco emissions. Conduct non-targeted analysis of emissions from new and emerging products.
**Data reporting**	Report data on a comparable metric across products (e.g. mass per total puff volume). Report data standardized to nicotine yield.
**Statistical power**	Balance statistical power with the length of the list of toxicants to compare across products. Use newly developed tools of risk assessment to prioritize toxicants for comparison.

## CHALLENGES OF COMPARISON

**Figure 1:**
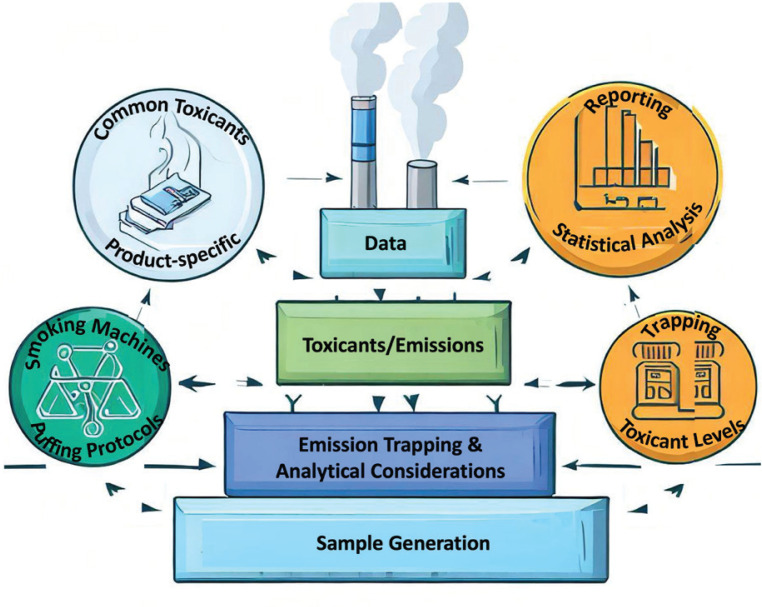
Challenges of comparing emissions between tobacco products

Figure 1 summarizes the challenges of comparing emissions between tobacco products and shows their interconnections that complicate the feasibility of head-to-head comparison. The following sections discuss these challenges in detail.

### Sample generation

*Smoking/vaping machines*

Smoking machines were designed to systematically generate and collect samples of tobacco product emissions for subsequent physical and chemical analysis^15,16^. However, the wide variability in the design and mode of operation of tobacco products, including cigars, cigarettes, ECs, and HTPs, has made the use of a one-size-fits-all smoking/vaping machine challenging. Commercial smoking machines are designed separately for different combustible tobacco product types where the smoking session is ended using a butt termination method. Vaping machines can sample ECs with and without puff-activating buttons for a specified number of puffs^17^. These sampling machines differ in their mode of operation, capacity, and range of puffing flow rates, thereby complicating a direct comparison of emissions from tobacco products^15^. Also, these machines have limited ability to connect to different shapes and geometries of product mouthpieces within and across categories of tobacco products (e.g. cigars and cigarillos of different dimensions, or different EC geometries and designs). Currently, some adaptors connect cylindrical-shaped tobacco products (i.e. cigarettes and cigars) to the available smoking machines. However, with tobacco product innovation, there is a need for the development of a leak-free, validated universal adaptor that can serve as a reliable interface between tobacco products of all mouthpiece geometries and smoking/vaping machines. Currently, tobacco control researchers rely on proprietary, custom-made mouthpiece adapters that are optimized for specific product geometries only (also provided by manufacturers upon request), raising concerns about the repeatability of the data generated using these adaptors^18,19^.

*Puffing protocols*

Puffing protocols used for sample generation of smoke/aerosol using smoking/vaping machines, are a major factor to consider in the comparison of emissions between tobacco products. These protocols give a description of the test criteria that should be followed during product testing, some of which include the puffing parameters (puff duration, frequency, airflow, and puff volume), pressure drop, product orientation, ventilation hole blocking (for cigarettes and ECs), product conditioning, and smoking environment (temperature and relative humidity). It is crucial that researchers include information on all these parameters in their study reports. Standard puffing regimes like the International Organization for Standardization (ISO), Massachusetts Department of Public Health (MDPH), and Health Canada Intense (HCI) regimes represent low, medium, and high-intensity regimes, respectively, that were developed for cigarette testing^14^. However, such standardized regimes do not reflect the complexity of the actual puffing of smokers^20^, including smokers’ puffing behavior changes in reaction to reduced nicotine delivery, a behavior known as compensation^21^, and a recent study provided additional evidence that these puffing regimes are not representative of the average smoker puffing behavior^22^. These cigarette regimes have been commonly used for aerosol generation from newer tobacco products^23-25^, and a standard puffing regime was recently introduced specifically for ECs and HTPs^26,27^. Since the actual use patterns influence the emission toxicants, besides product design and composition, using standard regimes to compare emissions across products could be misleading^28^. Developing product-specific puffing regimes that reflect the actual use of the various tobacco products would be useful, but this is complicated by different device designs in the same tobacco product category, which is especially apparent in ECs^28^.

Due to the limitations of applying standard puffing regimes to inhalable tobacco products, researchers have turned to replicate actual puffing regimes recorded from clinical studies, a technique known as ‘playback’^29,30^. In addition, researchers have modified the current cigarette standardized puffing regimes to fit other tobacco product designs. For instance, to allow the use of HCI and ISO for the generation of aerosols from Glo HTP, a research group modified the inter-puff interval for both HCI and ISO to 16s and 38s, respectively^31^. Inconsistencies in testing conditions between laboratories, and the lack of method validation, can render the replication of studies for product comparison challenging^29,32^.

Also, for cigarettes and cigars, the smoking session duration is determined by the butt length termination point. However, the end of aerosol generation sessions for other tobacco products like ECs and HTPs is variable. This is because ECs can produce a much higher number of puffs than combustible products in a single session, while various HTPs are designed with specific limits for the duration of a single-use session. The standardization of smoke/aerosol generation sessions is crucial for inter-product comparison of emissions, yet again this is complicated by the different operating principles of tobacco products and the variability within product categories^33^.

*ECIG/HTP manipulation and operation*

There are other considerations when generating samples from novel tobacco products. Some electronic tobacco products, for example, require preheating before aerosol production, which may impact emissions (e.g. IQOS)^34^. In addition, certain ECs require priming puffs or a higher flow rate to activate (e.g. Hyde EC), while others require pressing a button before inhalation (e.g. mod ECs)^2^. In addition, some ECs are customizable, allowing users to vary their operating parameters such as power, consequently altering the emission’s composition (also see ‘Product-specific toxicants/non-targeted analysis’ section below)^35^. Since the heat applied to aerosolize the EC liquid has such a strong relationship to the emissions produced, comparisons of EC products and combustible cigarettes, that have one mode of operation, are significantly complicated^36^.

Another important point to consider when comparing emissions from different tobacco products is the lack of standard reference products for some product types. For cigarettes, cigars, and cigarillos, standard products are representative of the most popular products in the US marketplace (e.g, 1R6F cigarettes)^37^. These standard products are critical in the development and validation of sampling methods and can qualify laboratory performance for cigarette emissions assessment. However, other tobacco products do not have standard reference products (e.g. ECs), which limits the ability to compare emissions generated by different laboratories.

### Emission trapping and analytical considerations

*Different techniques of aerosol trapping*

Emission trapping methods vary depending on the intended analyses, and conditions for optimal sample collection may vary across different product types. Capturing the particulate phase of machine-generated cigarette smoke on Cambridge filter pads is usually employed for quantifying non-volatile and semi-volatile constituents in mainstream smoke^38^. This approach is generally applicable for trapping the particulate matter of EC or HTP emissions. However, the saturation and effectiveness of the filter pad can be greatly affected by the mainstream aerosol. For example, EC aerosols quickly saturate filter pads due to high propylene glycol and glycerol content and their related hygroscopic properties^39^. This may lead to leakage from the filter pad and potential sample loss when the filter pad is removed from the holder. In addition, the retention of key EC aerosol constituents on the filter pads may be different from that in well-characterized methods developed for cigarette smoke analyses; for example, a fraction of the volatiles may dissolve in e-cigarette aerosol droplets and be collected by the pad. Accurate quantitation of many constituents that are present in EC emissions is more heavily undermined by the breakthrough of the particulate phase constituents from the filter pads because their levels are typically lower compared to those in cigarette smoke^40^. Similar considerations are relevant to the trapping of the gas phase components, which is usually done by using solvent-filled impingers or sorbent tubes. Approaches that have been developed and optimized for analyses of gas phase constituents in cigarette smoke, including large volumes of trapping solvents, will likely not be suitable to accurately quantify such constituents at lower, but still toxicologically relevant, levels in EC or HTP aerosols^40^. A variety of alternative approaches for collecting ‘unmodified’ EC aerosol condensate have been explored, such as using a set of connected modified pipet tips, a series of tracheal suction traps, or cold finger-trapping using liquid nitrogen^40-43^. While potentially effective, such methods would need to be analytically characterized and validated. Also, some aerosol constituents, like formaldehyde, can partition between particle and gas phases, and this should be taken into consideration in sampling tobacco products^31^.

*Analytical methods and matrix effect*

Analytical methods for constituent analyses in the trapped cigarette smoke fractions (i.e. particulate matter or gas phase) are also generally applicable to the analyses of these constituents in EC or HTP aerosols. However, the dynamic range and linearity of such methods, which have been optimized and validated for cigarette smoke, may not be suitable to accurately quantify lower or higher constituent levels found in EC and HTP aerosols. In addition, the aerosol matrix may affect analyte recoveries, especially when sample preparation involves complex sample purification procedures or derivatization. The matrix effect is also a factor in ion suppression, especially if constituents are analyzed by mass spectrometry-based techniques. Therefore, the use of identical, non-optimized analytical procedures for comparing constituent levels between cigarette smoke and EC or HTP aerosols, may produce inaccurate or misleading results.

Knowledge of the differences in the levels and identities of the toxicant emissions associated with various tobacco products informs the understanding of their relative health risks. Due to the differences in the characteristic chemical and physical properties of the emissions, whether smoke or aerosols, derived from cigarettes, cigars, hookah, ECs, and HTPs, the proper choice and application of a methodology require an understanding of its scope and limitations.

The most significant examples of incomplete information derived from tobacco product comparisons have involved the direct comparisons of EC versus cigarette emission profiles. Specifically, methods that are useful for characteristic cigarette smoke toxicants, such as formaldehyde, acrolein, acetaldehyde, and TSNAs have also been commonly employed for the evaluation of EC emission profiles. However, targeting selected cigarette toxicants in EC aerosols does not account for those compounds exclusive to ECs, such as the solvents propylene glycol and glycerol, or the numerous additives and flavorants, whose rigorous evaluation requires alternative analytical methodology^38^. Moreover, examples of characteristic and unique EC emissions include thermal degradation products of glycerol and propylene glycol^44,45^, specific additives^46^, flavorants and their reaction products with e-liquid solvents that exhibit enhanced toxicity compared to the parent flavorants^47,48^, and toxic transition metals specifically derived from EC components^49^. A strong example here is the exposure to vitamin E acetate during the EVALI crisis^50^.

*Physical assessment*

All inhalable tobacco products emit an aerosol of solid/liquid droplets (particulate phase) suspended in a gaseous phase. Hence, the particle size distribution is relevant to dosimetry and aerosol dynamics, and is a significant factor in determining the probability of particle deposition at different sites within the respiratory tract^51,52^. To date, particle size distribution has been studied mainly in ECs and cigarettes^53,54^. Each type presents characteristic analytical challenges. For example, cigarette smoke particle sizes are highly time- and environment-dependent. Fresh mainstream cigarette smoke consists of about 1000 particles/cm^3^, with the majority of the particles ranging between 0.1 and 1.0 μm in size^55,56^. Significant physical changes can occur upon ‘the aging’ of the smoke due to rapid coagulation and increases in humidity^57,58^. Particle growth due to water absorption occurs on the order of milliseconds. Cigarette smoke particle size does not change significantly as a function of cigarette type or smoking behavior^58^. In the case of ECs, a wide range of particle size measurements have been reported, attributed to the use of a variety of methods and instruments involving electrical mobility, inertial impaction, and light scattering^59^. Confounding factors in EC aerosol particle size measurements also include evaporation due to the high dilution ratios needed for many conventional instruments, as well as coagulation that occurs during the time between sampling and measurement. In addition, aerosol instrumentation such as cascade impactors and electrical mobility sizing systems, require steady-state flows^59^. The identity of variables that can significantly impact particle size distributions, such as EC design and resulting temperature, e-liquid ingredients, and puff topography, constitutes a significant current knowledge gap^60^.

### Toxicants/emissions

*Toxicant lists/targeted analysis – The FDA HPHC list as an example*

The targeted analysis of toxicants in new tobacco product emissions has focused on a relatively limited number of analytes or groups of analytes for direct comparison with historical data on cigarettes. This is due to factors including the accessibility of validated methodology and standards^61^. Importantly, there is also a critical lack of updated inhalation toxicity data to guide the prioritization of targets^62^. In 2012, the US FDA published a list of 93 harmful and potentially harmful constituents (HPHCs) in tobacco products and tobacco smoke, based on available toxicity data for priority assessment of the risks of tobacco product emissions^63^, and further published a guidance document for industry on which HPHCs to focus on for reporting (20 for cigarette smoke, 9 for smokeless tobacco, 6 for roll-your-own tobacco)^64^. However, a wide variety of new tobacco products have been introduced into the US and global market since 2012. In addition to a needed update of the HPHC list to include toxicant emissions characteristic of the new electronic products and formulations, toxicity data are also needed that facilitate the comparison of emissions and relative risks of various products^65^. In 2019, the US FDA called for comments to update the established HPHC list to reflect the emergence of novel tobacco products such as ECs, which were not previously covered. However, to date, the list remains the same as in 2012^66^. Other regulatory agencies have adopted lists with even fewer toxicants, such as the WHO Framework Convention on Tobacco Control (FCTC), which contains 38 toxicants on a priority list recommended for mandated lowering^67^. In addition to the need for updating the number of HPHCs to account for novel tobacco products, the incomplete listings also highlight the significant current lack of inhalation toxicity data, particularly for new tobacco product emissions. More research is needed to generate and update the inhalation toxicity data of several constituents that are not yet on the FDA HPHC list and similar lists.

*Product-specific toxicants/non-targeted analysis*

While toxicants in cigarettes and cigarette smoke are well characterized – a fact that is reflected in the mentioned HPHC priority lists by the US FDA, the WHO FCTC, and others, such clarity is lacking for novel tobacco products such as HTPs and ECs. In HTP products, tobacco is not burnt but rather heated in the device, resulting in a similar toxicant profile compared to cigarettes (albeit at lower levels), with one exception: humectants may be present to facilitate aerosol generation^68^. However, one study detected an HTP-specific toxicant in IQOS emissions^69^.

ECs differ substantially from combustible and HTP products in that they usually do not contain tobacco leaves, but rather a nicotine solution in propylene glycol and glycerol, along with a wide variety of additives intended to regulate pH, impart flavor, and other functions^2^. ECs are also unique in the manipulation of nicotine parameters in their e-liquids: nicotine concentration, nicotine form (freebase or salt), and nicotine source (tobacco-derived or synthetic)^70,71^. As a result, while some tobacco-specific toxicants are absent in EC aerosol, thousands of chemical compounds have been detected in EC aerosol^72^, and toxicants including listed HPHCs such as carbonyls (e.g. acetaldehyde, acrolein, and formaldehyde) continue to be of concern^73^. This is further complicated by the fact that EC power output and resulting temperature, as well as the presence of certain solvent and flavor constituents, play a role in the quantities of HPHCs produced^74^. Evidence is emerging that certain food-safe flavorants used in e-liquids such as diacetyl (butter flavor), or cinnamaldehyde (cinnamon) are harmful when inhaled^75^. The proposed 2019 update to the FDA HPHC list mentioned above contains several flavorants such as diacetyl and isoamyl acetate alongside the common e-liquid solvents propylene glycol and glycerol as respiratory toxicants^65^. In addition, there is evidence that some of the most popular flavorants including vanillin, ethylvanillin, and benzaldehyde undergo chemical reactions with e-liquid solvents during storage, resulting in novel compounds with concerning toxicological profiles^47,48^. Also, the metal and ceramic heating coils used in ECs can release ultrafine droplets and a variety of problematic metals and metalloids into the aerosol that could be specific to ECs^76,77^.

Lastly, the mode of operation of ECs and HTPs can lead to different chemical profiles of emissions compared to cigarettes^78^. This necessitates the use of non-targeted analyses to comprehensively characterize the emissions of these novel products, an approach that has been reported recently^72,79^, to more fully understand the toxicity of the emissions from tobacco products. More industry-independent non-targeted analysis studies are needed to fully characterize the emissions of new tobacco products^80^.

### Data reporting

*Mass per item, session, puff, or nicotine yield*

For data reporting, there is no consensus if emissions should be reported as absolute mass, mass per product tested, mass per puff, mass per nicotine dose, or inhaled concentration when comparing emissions across inhalable tobacco products. Reporting absolute mass is the least informative while mass per item (e.g. cigarette) is not applicable to some tobacco products like ECs. Also, mass per puffing session is not sufficient, especially when products with wide variability in the number of puffs per session are compared (e.g. hookah versus cigarette). Mass per puff overcomes technical challenges and use modes of different products, allows for a direct comparison of emissions between products, and could be easily scaled up to estimate daily exposure^81^. However, inter-product variability in puff parameters questions the validity of this metric as these parameters may affect the toxicant intake^29,32^. An alternative approach for data reporting could be mass per total puff volume (flow rate × puff duration × the number of puffs) which allows a comparison between tobacco products even with different puffing parameters and session lengths.

Nevertheless, some researchers argue that whatever data reporting approach one follows, there is a need to report emissions normalized by nicotine yield to account for nicotine self-titration among user^82^. In a study comparing free radical emissions from HTPs to ECs and a reference combustible cigarette, the authors showed that yield per puff allowed for a direct comparison of emissions from these different products^82^. However, the same report showed that when the results were normalized by nicotine yield emitted from each product, the order of increased emissions was reversed for some products (e.g. the Ploom EC hybrid device or the SREC reference EC compared to HTPs; figure 1 in Bitzer et al.^82^). This report noted that the samples were generated using a single puffing regime, and different representative regimes of actual use of these products may result in different trends. Another group argued that nicotine yield does not matter exclusively, but nicotine emission rate as well (i.e. nicotine flux: µg/s). Nicotine flux was compared across tobacco products including combustibles (e.g. cigarettes, cigars, and hookah) and electronic products like ECs and HTPs^83^. This comparison showed that products may be equivalent in terms of total nicotine yield, yet have different nicotine emission rates due to different puffing topography (i.e. delivery of the same amount of nicotine over varying amounts of time). Hence, it could be useful to compare toxicant flux between products as well.

### Statistical analysis considerations

One more consideration of data reporting relates to the statistical equivalence or difference between tobacco products. Two given products are equivalent if no statistically significant differences exist for all toxicants considered, yet these products would be considered different if a statistically significant difference exists for only one of several toxicants. Hence, if emissions from different tobacco products are compared, it is necessary to select constituents in emissions with available toxicity data, yet the list to be compared should not be exhaustive to ensure good statistical power. Approaches have been examined in the literature to determine what emissions should be prioritized for reduction and hence comparison between tobacco products. For example, the threshold of toxicological concern (TTC) is designed to assess constituents with known structural information, but for which toxicological information is lacking^84^. Also, toxicological modeling like quantitative risk assessment (QRA) could be powerful to screen the chemical profile of emissions from different tobacco products^85^.

## CONCLUSION

We have discussed the challenges of comparing emissions from tobacco products covering sample generation, emission trapping, analytical method considerations, toxicant prioritization for monitoring, and data reporting and statistical analysis challenges (Table 1). However, even if the challenges to standardizing a comparison methodology between tobacco product emissions are addressed, this approach should be considered with caution for at least three reasons. First, a tobacco product could be shown to emit fewer and lower toxicants than another product, but this does not readily translate into lower risk, largely because the acute and longer-term inhalation toxicity of each component must be considered; and the paucity of these data constitutes an important data gap. Second, the paradigm of comparing emissions between newer tobacco products and traditional cigarettes often obscures the more meaningful comparison to inhaling clean air. As public health researchers, we must shift the focus from the relative to the absolute toxicity of a given product, to make the risks of using these products clearer. Third, because humans can differ widely in using different tobacco product types (including poly tobacco use), comparison of machine-smoked emissions across products should only be used as a first measure of product evaluation by scientists and regulators. Nevertheless, multidisciplinary collaborations between emission testing experimentalists, behavioral scientists, clinicians, and policymakers will ensure that comparing tobacco products is kept in context, validated, and does not readily translate to comparing risk. The scientific community and the regulatory agencies (e.g. US FDA) make clear distinctions between reduced exposure and reduced risk. This needs to be clearly communicated to consumers that are targeted by tobacco industry marketing that could use exposure comparison to imply risk reduction without substantial and long-term evidence. Regulatory agencies need to monitor the market closely to prevent marketing that leads to risk misperceptions.

## Data Availability

Data sharing is not applicable to this article as no new data was created.
